# *Ehrlichia chaffeensis* TRP32 Nucleomodulin Function and Localization Is Regulated by NEDD4L-Mediated Ubiquitination

**DOI:** 10.3389/fcimb.2017.00534

**Published:** 2018-01-11

**Authors:** Tierra R. Farris, Bing Zhu, Jennifer Y. Wang, Jere W. McBride

**Affiliations:** ^1^Departments of Microbiology and Immunology, University of Texas Medical Branch, Galveston, TX, United States; ^2^Pathology, University of Texas Medical Branch, Galveston, TX, United States; ^3^Cell Biology, University of Texas Medical Branch, Galveston, TX, United States; ^4^Center for Biodefense and Emerging Infectious Diseases, University of Texas Medical Branch, Galveston, TX, United States; ^5^Sealy Center for Vaccine Development, University of Texas Medical Branch, Galveston, TX, United States; ^6^Institute for Human Infections and Immunity, University of Texas Medical Branch, Galveston, TX, United States

**Keywords:** Ehrlichia, ubiquitination, post-translational modification, effector, NEDD4L, localization, tandem repeat protein

## Abstract

*Ehrlichia chaffeensis* is an obligately intracellular bacterium that reprograms the mononuclear phagocyte through diverse effector-host interactions to modulate various host cell processes. In a previous study, we reported that the *E. chaffeensis* nucleomodulin TRP32 regulates transcription of host genes in several biologically relevant categories, including cell differentiation and proliferation. In this study, we investigate the effect of ubiquitination on TRP32 function and localization within the host cell. TRP32 is both mono- and polyubiquitinated on multiple lysine residues during infection and when ectopically expressed. Despite lacking a canonical PPxY motif, TRP32 interacted with, and was modified by the human HECT E3 ubiquitin (Ub) ligase NEDD4L. TRP32 ubiquitination was not by K48-linked polyUb chains, nor was it degraded by the proteasome; however, TRP32 was modified by K63-linked polyUb chains detected both in the cytosol and nucleus. HECT ligase inhibitor, heclin, altered the subnuclear localization of ectopically expressed TRP32 from a diffuse nuclear pattern to a lacy, punctate pattern with TRP32 distributed around the periphery of the nucleus and nucleoli. When a TRP32 lysine null (K-null) mutant was ectopically expressed, it exhibited a similar phenotype as single lysine mutants (K63R, K93R, and K123R). However, the K-null mutant showed increased amounts of cytoplasmic TRP32 compared to single lysine mutants or heclin-treated cells ectopically expressing TRP32. These alterations in localization corresponded to changes in TRP32 transcriptional repressor function with heclin-treated and single lysine mutants unable to repress transcription of a TRP32 target genes in a luciferase assay.

## Introduction

*Ehrlichia chaffeensis* is a gram-negative, obligately intracellular bacterium and the etiologic agent of human monocytotropic ehrlichiosis (HME), an emerging life-threatening tick-borne zoonosis. In humans, *E. chaffeensis* preferentially infects mononuclear phagocytes, causing an acute infection that manifests as an undifferentiated febrile illness. The mechanisms by which *E. chaffeensis* reprograms various host cell processes is not fully understood; however, a group of type 1 secreted, tandem repeat protein (TRP) effectors similar to the repeats-in-toxin family of exoproteins are involved. TRPs were initially identified as *E. chaffeensis* major immunoreactive proteins, and are known to elicit protective antibody responses (Kuriakose et al., 2012). Recent studies have revealed that TRPs are secreted pleotropic effectors that interact with a large group of functionally diverse host cell proteins as well as host cell DNA (Lina et al., 2016b).

The most well-characterized TRP effectors, TRP120 and TRP32, interact with many host cell targets, directly activate cell signaling pathways, and activate/repress host cell transcription. Surface-expressed TRPs contribute to ehrlichial entry via WNT pathway activation (Luo et al., 2015). Additionally, TRP120 interactions with ADAM17 on the host cell surface activate the Notch pathway, resulting in the downregulation of innate immune toll-like receptors (Lina et al., 2016a). TRP120 and TRP32 also act as nucleomodulins that manipulate host gene transcription via direct interactions with host target genes. TRP120 binds a GC-rich motif, leading to upregulation of specific host genes involved in transcriptional regulation, signal transduction, and apoptosis (Zhu et al., 2011). TRP32 also binds a G-rich motif consisting of imperfect GGTGGC-like sequence repeats, but preferentially targets genes regulating cell proliferation and differentiation. TRP32 was also shown to activate and repress expression of targets in a gene-specific manner during infection and in a luciferase reporter assay (Luo and McBride, 2012; Farris et al., 2016).

A common theme among bacterial pathogens is the hijacking of host post-translational machinery to modify effectors (Ribet and Cossart, 2010; Ravikumar et al., 2015; Popa et al., 2016). *E. chaffeensis* effectors are phosphorylated, ubiquitinated, and SUMOylated by host enzymes (McBride et al., 2011; Wakeel et al., 2011; Dunphy et al., 2014; Farris et al., 2016; Zhu et al., 2017), and these PTMS are important for effector function. TRP120 ubiquitination and SUMOylation is required for interactions with host proteins, and TRP32 tyrosine phosphorylation plays a role in its nuclear localization.

Ubiquitination is the covalent attachment of the small peptide modifier ubiquitin (Ub) that occurs via an enzymatic cascade requiring the sequential action of three classes of enzymes the third of which, the E3 Ub ligase (~600 known), determines substrate specificity (Metzger et al., 2012). Ubiquitination occurs either singly (monoubiquitination) or as a chain covalently linked via any one of seven Ub lysine residues or attached to the N-terminus. All homotypically-linked chains as well as heterotypic and branched polyUb chains have been detected in cells, which direct the substrates to different fates within the cell (Ub and Ub-like proteins as multifunctional signals). The most studied Ub modifications are K48-linked chains which direct proteasomal degradation of target substrates, K63-linked chains which are involved in cell signaling, receptor endocytosis, and protein-protein interactions, and K11-linked chains which may play a role in cell cycle-specific protein degradation (Komander, 2009).

Although bacteria do not possess an endogenous Ub system, the interaction of bacterial effectors with the ubiquitin system is well-described with bacterial proteins acting as both Ub substrates and ligases (Thomas and Holden, 2009; Collins and Brown, 2010). When bacterial proteins are modified by ubiquitin, these modifications can influence both physical and temporal localization of the protein depending on whether the Ub conjugate is a single Ub or a polyUb chain. Monoubiquitination typically alters protein localization and is involved in both nuclear import and export of eukaryotic proteins (Salmena and Pandolfi, 2007). *Salmonella* is known to exploit ubiquitination to alter subcellular localization of the SopB effector (Patel et al., 2009). SopB is a phosphatase that first localizes to the host membrane, where its enzymatic functions alter actin organization, facilitating bacterial entry. After SopB is multiply monoubiquitinated, it trafficks to the bacteria-containing vacuole where it alters vesicular trafficking to facilitate bacterial replication (Anderson and Frank, 2012). The functional equivalent of polyubiquitination is typically dependent on chain type. However, one of the best characterized examples is the *Legionella* effector SidH, which utilizes K48-linked polyubiquitination for temporal regulation (Ashida et al., 2014).

Although examples of pathogen effectors with PTM-associated functions exist, ehrlichial effector regulation by ubiquitination has previously only been described for TRP120 in which ubiquitination has been linked to protein-protein interactions (Zhu et al., 2017). In this study, we examined the role of ubiquitination on TRP32 transcription factor function. We demonstrate that multiple species of mono- and polyubiquitinated TRP32 can be detected during infection, and that the host E3 enzyme, NEDD4L, ubiquitinates TRP32. Moreover, we found that these Ub modifications are required for TRP32 transcription factor function and subnuclear localization.

## Materials and methods

### Cell culture and infection

*Ehrlichia chaffeensis* (Arkansas strain) was propagated in a human monocytic cell line (THP-1; ATCC, Manassas, VA). THP-1 cells were maintained in RPMI 1640 with HEPES (25 mM) (Invitrogen; Carlsbad, CA) supplemented with 10% fetal bovine serum (FBS)(HyClone, Logan, UT), 5 mM L-glutamine, 1% sodium pyruvate, and 12.5 ml 10% glucose (Sigma; St. Louis, MO) at 37°C in a 5% CO_2_ atmosphere. *E. chaffeensis* infection was maintained by subculturing infected cells with uninfected. HeLa cells (human cervical epithelial; ATCC) for transfection were grown in MEM (Invitrogen) supplemented with 10% FBS (HyClone) and maintained in a 5% CO_2_ atmosphere. When stated cells were incubated with 10 mM bortezomib (ApexBio; Houston, TX), PYR41 (Thermo Fisher; Boston, MA), Heclin (Sigma), carfilzomib (ApexBio) or vehicle (DMSO) for 12–24 h before harvesting cells for lysate or luciferase expression measurement.

### Expression and purification of recombinant TRP32

Full-length TRP32 was PCR amplified from *E. chaffeensis* genomic material and cloned into pGEX-6p1 vectors (GE Healthcare; Piscataway, NJ). The constructs were transformed into BL21 *E. coli* (Genlantis; San Diego, CA) for protein expression. Briefly, overnight cultures were diluted 1:20 in LB plus ampicillin (Amp) and grown for 3 h with agitation at 37°C then protein expression was induced by adding isopropyl-β-D-thiogalactoside (IPTG) to a final concentration of 0.5 mM and growing for another for 3–4 h at 37°C. Cells were then suspended in Tris-buffered saline with protease inhibitors (cOmplete mini, EDTA free) (Sigma), lysed by sonication, then cleared by centrifuging for 20 min at 12,000 g at 4°C. Cleared lysate was then added to washed Glutathione Sepharose 4B (GE) and recombinant proteins were purified according to the manufacturer's instructions.

### *In Vitro* microfluidic peptide ubiquitination array

Peptides (12-mer) corresponding to TRP32 putative ubiquitination targets were synthesized by flanking a central lysine residue with 6 N-terminal amino acids and 5 C-terminal amino acids. For each peptide, a corresponding negative control sequence was included with alanine (A) substituted in place of K (A peptide). Peptides were synthesized on chip and an *in vitro* ubiquitination assay performed as previously described (Zhu et al., 2016). Briefly, ubiquitination reactions were performed using an *in vitro* ubiquination kit (Enzo Life Sciences; Farmingdale, NY) in the presence of THP-1 lysate. Nonspecifically bound proteins were removed by washing with buffer containing 1% SDS, 0.1% β-mercaptoethanol and 100 mM Tris for 30 min, followed by a PBS wash containing 1% Tween-20 (PBST) then Ub was detected using anti-Ub Alexa Fluor 647 antibody (Santa Cruz Biotechnology, 1:300). The signal was then read using an Anon GenePix 4400A (Molecular devices) scanner running GenePix Pro 7 software. Positive signals were determined by comparison with the control peptides and negative controls for each assay. Differences between wild-type and control peptides were assessed using two-tailed Student's *t*-test. The data are presented as mean ± SD, and significance was indicated by a *p* < 0.05.

### *In Vitro* ubiquitination assay

TRP32 ubiquitination was performed with recombinant TRP32 and an *in vitro* ubiquitination kit (Enzo Life Science). Briefly, TRP32 (200 nM) was added to ubiquitination buffer, biotin-tagged Ub protein, E1 ligase and the E2 ligase UBCH5b with and without the E3 NEDD4L and Mg-ATP. Reaction mixtures were incubated at 30°C for 3 h and then boiled with 1x lithium dodecyl sulfate (LDS) sample buffer. Samples were separated by SDS-PAGE and proteins detected by Western blotting with rabbit anti-TRP32 (1:10,000) or anti-Ub (FK2, Enzo, 1:1,000) primary antibodies followed by horseradish peroxidase-conjugated anti-rabbit or anti-mouse secondary antibodies (1:10,000) (KPL; Gaithersburg, MD). Bound antibodies were visualized after incubation with ECL substrate (Pierce; Rockford, IL).

### Cotransfection and HA immunoprecipitation

HeLa cells were cotransfected with pAcGFP1-C or pAcGFP1-TRP32, and WT or lysine null HA-tagged Ub constructs by use of Lipofectamine 2000 (Invitrogen). Cells were collected and lysed in 20 mM Tris, pH 7.5, 150 mM NaCl, 1 mM EDTA, 20 mM NEM (covalent isopeptidase inhibitor; Sigma), 1% Triton X-100, and protease inhibitors (Complete mini, EDTA-free; Sigma) at 24 h post-transfection. Lysates were then centrifuged at 4°C and 16,000 g (Eppendorf 5430R centrifuge with model FA 45-30-11 rotor) for 20 min, and the supernatants were incubated with anti-HA agarose (Thermo Scientific) overnight at 4°C. The resin was washed with lysis buffer, boiled in LDS buffer, and analyzed by SDS-PAGE and Western blotting.

### Antibody crosslinking and immunoprecipitation

Rabbit anti-TRP32 and control pre-immune serum were crosslinked to Protein A/G Plus Agarose using the Pierce Crosslink IP kit according to manufacturer's instructions (Luo et al., 2008). Briefly, 20 ug of antibody was incubated with washed A/G resin for 1 h at room temperature. Resin was then washed with PBS to remove unbound antibody. The chemical crosslinker disuccinimidyl suberate (DSS) was added and sample was incubated again for 1 h at room temperature. Resin was then washed 3x with elution buffer, then 2x in wash buffer to remove un-crosslinked antibody. Resin was then stored at 4° in PBS until use. For immunoprecipitation, lysate was harvested from heavily infected THP-1 cells. Cells were pelleted at 4° C and washed 2x with ice cold PBS. Then cells were resuspended in lysis buffer (TBS with 1% Triton X-100, cOmplete protease inhibitor (Sigma), 1 mM phenylmethylsulfonylflouride, 1 mM ethylenediaminetetraacetic acid, and 50 mM N-ethylmaleimide) and incubated for 30 min on ice, vortexing for 30 s every 5 min. Lysate was then centrifuged at 12,000 g for 15 min to clear and soluble fraction retained. Lysate was then pre-cleared by incubating for 30 min with control agarose resin. Cleared lysate (500 to 1,000 μg) was added to TRP32 and control resin and incubated, on a rotator, at 4° C overnight. Resin was then washed 4–6x with lysis buffer and then immunoprecipitated proteins were eluted in 50 μl elution buffer (Pierce). Ubiquitin enrichment was performed using a Ubiquitin Enrichment kit (Pierce) according to manufacturer's instructions.

### Localization

TRP32 lysine mutants were created from TRP32 in a pAC-GFP-CI (Clontech; Mountain View, CA) backbone using a QuikChange Mutagensis II kit (Agilent; Santa Clara, CA), or were obtained from a commercial vendor (GenScript). Mutants were amplified in XL1-Blue *E. coli* and purified using the PerfectPrep EndoFree Plasmid Maxi Kit (5 Prime). Purified plasmids were transfected into HeLa cells using Lipofectamine 2000 (Thermo Fisher) per manufacturer's instructions. Cells were acetone fixed and mounted with ProLong Gold Antifade reagent with DAPI (4′,6-diamidino-2-phenylindole) (Invitrogen). Localization of ectopically expressed constructs was examined at 24 h post-transfection using immunofluorescence microscopy and confocal microscopy. For immunofluorescence microscopy, cells were incubated with mouse anti-B23 (1:100)(Santa Cruz; Santa Cruz, CA) or mouse anti-coilin (1:100)(Santa Cruz) for 1 h, then washed and incubated with Alexa Fluor 594-IgG(H+L) labeled goat anti-mouse secondary antibody (1:100) (Molecular Probes; Carlsbad, CA) for 1 h then mounted with ProLong Gold Antifade reagent with DAPI (Invitrogen). For confocal HeLa cells were seeded unto a glass cover slip, TRP32-GFP expression constructs were transfected (Lipofectamine 2000) and cells harvested 24 h post-transfection and fixed in 4% paraformaldehyde in PBS for 20 min at room temperature. Cells were then permeabilized using 1% Triton X-100 with 5% bovine serum albumin (BSA) in PBS for 1 h. After permeabilization, cells were incubated with rabbit anti-TRP32 (1:1000) for 30 min, then washed and labeled with Alexa Fluor 488-IgG(H+L) conjugated goat anti-rabbit secondary antibodies (1:100; Molecular Probes) for 30 min before mounting with ProLong Gold antifade reagent with DAPI (4′,6-diamidino-2-phenylindole) (Invitrogen). Samples were examined using a Zeiss LSM 510 meta laser scanning confocal microscope configured with an Axiovert 200M inverted microscope using a c-Apochromat 63x/1.2 numerical aperture water immersion lens. UV argon, visible argon ion and green helium neon lasers were used and emissions read using 385–470 nm (DAPI), 505–530 nm (Alexa Fluor 488-conjugate), 560–615 nm (Alexa Fluor 594-conjugate) band pass filters, respectively. Images were analyzed with LSM 510 software and Z-stacks were constructed by imaging optical slices at 1 μM intervals. FIJI was used for subsequent image processing and only linear adjustments (i.e., brightness, contrast) were made (Schindelin et al., 2012).

### Luciferase gene expression assay

Promoter regions containing the TRP32 binding sites as determined using the Genomic Regions Enrichment of Annotations Tool (GREAT) were cloned into the promoterless firefly luciferase vector pGL4.10 (Promega; Madison, WI)(McLean et al., 2010). Each promoter construct (200 ng) was transfected into HeLa cells using Lipofectamine 2,000 (Thermo Fisher) along with GFP-TRP32, or an empty GFP expressing vector and a control vector expressing *Renilla* luciferase under the control of a HSV-thymidine kinase promoter (pRL-TK Vector, Promega). Equal amounts of total DNA were transfected into each cell. After 24 h, cells were harvested and Dual Glo Luciferase Reagent (Promega) added to the cells according to the manufacturer's protocol. Relative light output was measured using a Veritas Microplate Luminometer. Relative expression levels were calculated for each gene compared to the control using a Student's *t*-test.

## Results

### TRP32 is mono- and polyubiquitinated at multiple residues

**Figure 1:**
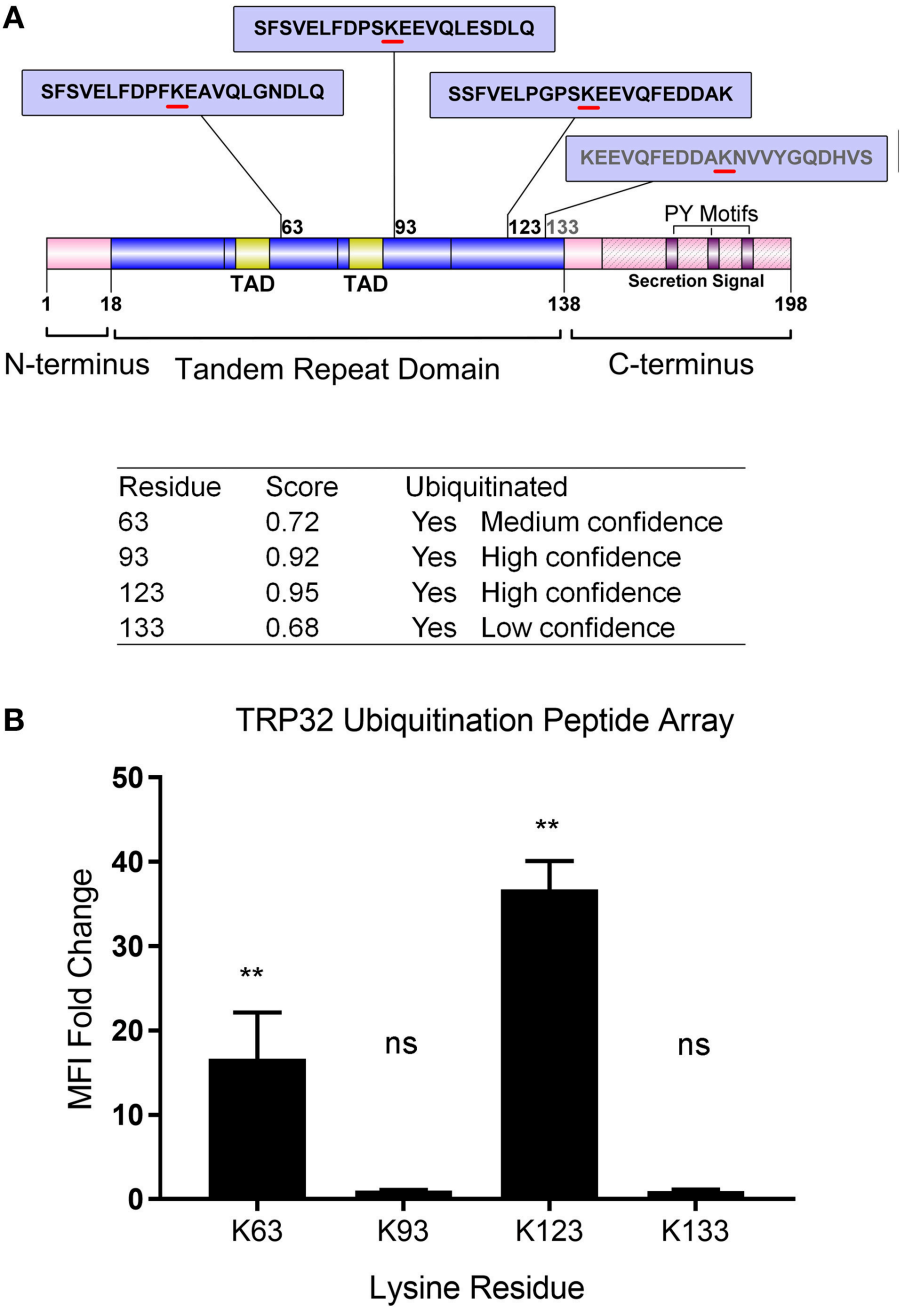
Schematic of *E. chaffeensis* TRP32 domains and predicted sites of ubiquitination and *in vitro* ubiquitination of TRP32 lysine residues using a peptide array. **(A)** TRP32 contains four lysine residues, all of which are located within the tandem repeat domain and are predicted to be ubiquitinated with varying confidence levels. Proline-Tyrosine (PY) motifs: TPYY(165-168), NPYY(176-179), TPDY(185–188) are also shown. **(B)** TRP32 lysine containing peptides were tested in a microfluidic peptide ubiquitination assay. Lysine 63 and 123 showed significantly higher signals than corresponding peptides in which the lysine residues were substituted with alanine.

**Figure 2:**
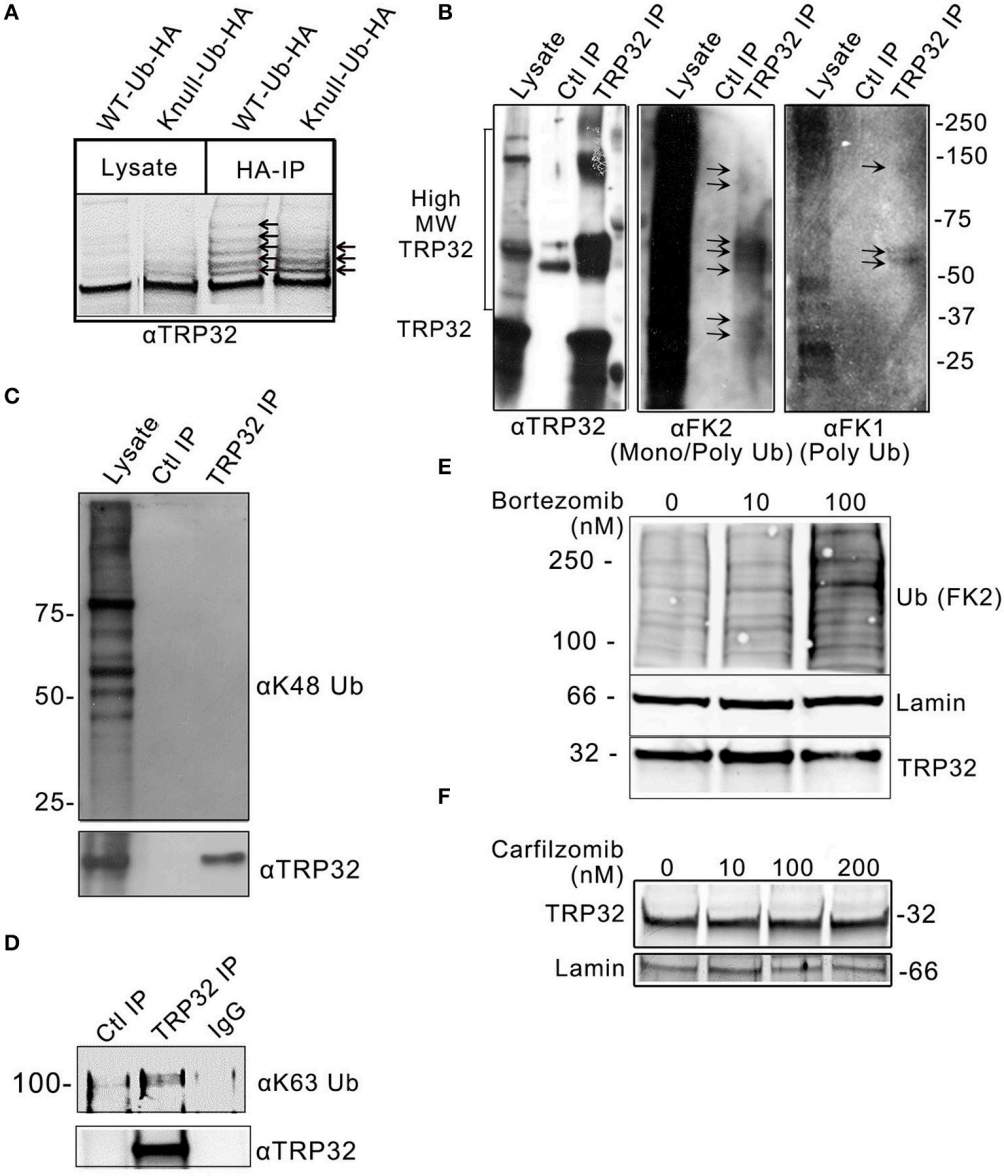
*E. chaffeensis* TRP32 is mono and polyubiquitinated, but is not degraded by the proteasome. **(A)** GFP-tagged TRP32 was cotransfected into HeLa cells with HA-tagged WT and K-null Ub constructs. HA-immunoprecipitation was performed and the resulting eluate was probed with anti-TRP32 specific antibody by immunoblot. Multiple higher molecular weight bands indicative of ubiquitinated species of TRP32 were detected (arrows). **(B)** Multiple species of ubiquitinated TRP32 were also detected during infection of THP-1 cells. Anti-TRP32 specific antibody was used to immunoprecipitate TRP32 from infected THP-1 lysate. The resultant immunoprecipitated protein was probed with antibodies specific for conjugated mono and polyUb chains (FK2) and for polyUb chains alone (FK1) by immunoblot. Bands were detected (arrows) between 25 and 37 kDa, 50 and 75 kDa, and above 100 kDa that colocalized with TRP32 bands. Immunoprecipitation of TRP32 and immunoblot with anti-K48 and anti-K63 Ub antibodies show absence of K48 ubiquitination **(C)** and presence of K63-linked polyubiquitinated TRP32 **(D)**. TRP32 levels were unaffected by treatment with a proteasome inhibitor. *E. chaffeensis-*infected cells were incubated with varying concentrations of the proteasome inhibitor bortezomib **(E)** for 12 h or carfilzomib **(F)** for 8 h prior to harvest and immunoblotting with anti-TRP32 and anti-lamin (ctrl) and anti-Ub (FK2) antibodies.

*In silico* analysis identified multiple lysine residues in TRP32 that were predicted to be sites of ubiquitination, two of which were proximal to predicted transcriptional transactivation domains (TAD) (Figure 1A; Radivojac et al., 2010). When 12-mer peptides corresponding to putative TRP32 ubiquitination sites were examined *in vitro* using a microfluidic peptide array for their ubiquitination potential, two sites were identified (Figure 1B). In order to determine if TRP32 was ubiquitinated in human cells, we ectopically expressed GFP-tagged TRP32 along with HA-tagged wildtype (WT) or K-null Ub and performed HA-immunoprecipitation. Unmodified TRP32 migrates at approximately 32 kDa, higher than expected due to its low pI (3.44) and hydrophilicity (Luo et al., 2008; Shirai et al., 2008). Multiple high molecular weight TRP32 bands were observed when ectopically expressed TRP32 was co-precipitated with HA-tagged WT or K-null Ub that is unable to form polyUb chains. Fewer high molecular weight bands were observed in the K-null Ub compared to the WT Ub pulldown (Figure 2A). The presence of multiple high molecular mass bands in the K-null Ub sample suggests that TRP32 is modified on multiple lysine residues while the presence of additional higher molecular mass bands in the WT Ub pulldown indicated TRP32 polyubiquitination. Next, we examined TRP32 ubiquitination during infection by performing an IP using a TRP32-specific antibody or a serum control and probing the eluate with antibodies specific for polyUb (FK1) and mono- and polyUb conjugates (FK2). Ub-positive bands that coincided with detection of TRP32 (Figure 2B, panel 1) were detected at ~37, 40, ~60, and ~100 kDa by the FK2 antibody (mono- and polyUb) (Figure 2B, panel 2); polyubiquitinated TRP32 was detected at ~60 and 100 kDa by the FK1 antibody (polyUb specific; Figure 2B, panel 3). These findings are consistent with the presence of both multi-mono and polyubiquitinated TRP32. When the eluate was further probed using an antibody specific for K48-linked polyUb chains, none were detected (Figure 2C); however, K63Ub was detected on TRP32 (Figure 2D). Further, TRP32 did not appear to be degraded by the proteasome as treatment with varying concentrations of the proteasome inhibitors carfilzomib (IC50 < 5 nM) and bortezomib (IC50 < 10 nM) did not alter TRP32 proteins levels (Figures 2E,F). These findings support the conclusion that the polyUb chains decorating TRP32 are not canonical K48-linked chains.

### TRP32 is ubiquitinated by NEDD4L with K63-linked PolyUB

**Figure 3:**
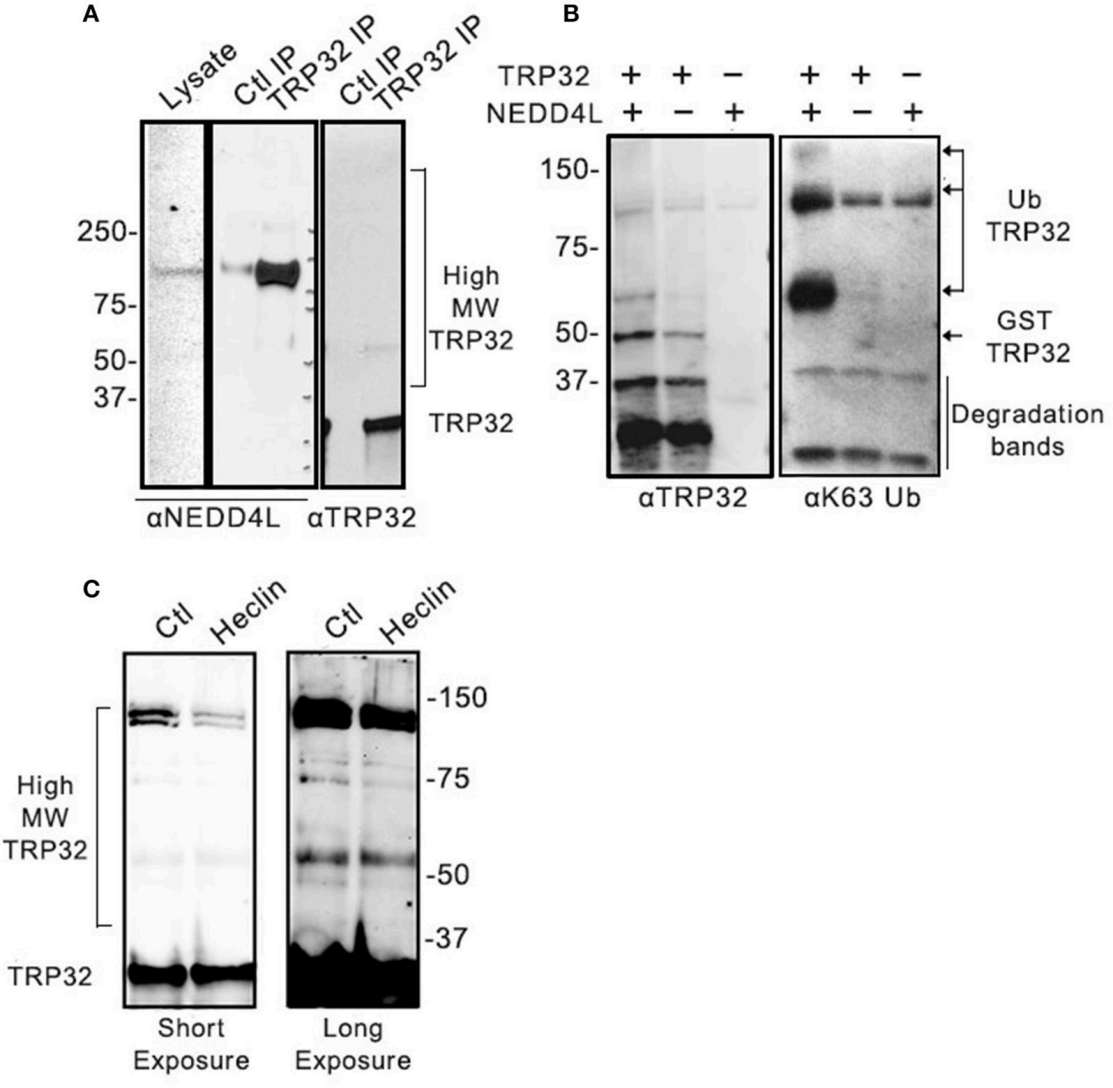
Host NEDD4L E3 ligase conjugates TRP32 with K63-linked polyUb chains. **(A)** Immunoprecipitation of NEDD4L with anti-TRP32 demonstrating interaction in *E. chaffeensis-*infected cells. The resulting eluate was probed with anti-NEDD4L and anti-TRP32 antibody by immunoblot. **(B)** NEDD4L conjugates K63-linked polyUb chains to TRP32 *in vitro*. An *in vitro* assay was performed in the presence or absence of NEDD4L and the resultant reaction was immunoblotted with anti-TRP32 and anti-K63-linked polyUb-specific antibodies. Bands corresponding to K63 linked polyubiquitinated TRP32 (arrow) were increased in the presence of NEDD4L. **(C)** Treatment with the E3 ligase inhibitor, heclin, decreased TRP32 higher molecular weight bands. *E. chaffeensis*-infected THP-1 cells were incubated with heclin (25 uM) for 12 h before harvesting lysate and immunoblotting with anti-TRP32 specific antibodies.

We have demonstrated that NEDD4L is a HECT E3 Ub ligase that is upregulated during infection, colocalizes with ehrlichial morulae, and interacts and ubiquitinates TRP120 (Zhu et al., 2017). We examined TRP32 and identified multiple motifs that resemble the proline-rich NEDD4L interaction motif. Therefore, we examined the role of NEDD4L in TRP32 ubiquitination. To determine if TRP32 and NEDD4L interact during infection, immunoprecipitation was performed on *E. chaffeensis-*infected THP-1 cell lysate using either an anti-TRP32 antibody or serum control, and the resulting eluate was probed with anti-NEDD4L antibody. NEDD4L precipitated with TRP32, suggesting an interaction during infection (Figure 3A). To investigate this further, we performed an *in vitro* ubiquitination assay on recombinant GST-tagged TRP32 in the presence and absence of recombinant NEDD4L. We found an increased abundance of high molecular weight isoforms of TRP32 when NEDD4L was included (Figure 3B, left panel). Because NEDD4L primarily catalyzes the linkage of K63-linkaged polyUb chains, we probed the *in vitro* ubiquitination reactions with an anti-K63 linkage-specific antibody. We observed a strong band at ~60 kDa, and a fainter band above 150 kDa by Western immunoblot that were consistent in mass to bands detected in the TRP32 immunoblot, demonstrating that NEDD4L was able to catalyze TRP32 K63-linked polyubiquitination (Figure 3B, right panel). Additionally, when *E. chaffeensis-*infected cells were treated with heclin, an inhibitor of HECT family ligases including NEDD4L, some higher molecular weight isoforms of TRP32 (~45 and ~130 kDa) were decreased compared to untreated cells, suggesting NEDD4L ubiquitination of TRP32 (Figure 3C). Notably, the greatest decreases were seen in the bands that appear to correspond to bands (~65 and 160 kDa) in GST-tagged TRP32 in the NEDD4L *in vitro* assay shown in Figure 3B.

### Ubiquitination of TRP32 effects subcellular localization and transcription factor function

**Figure 4:**
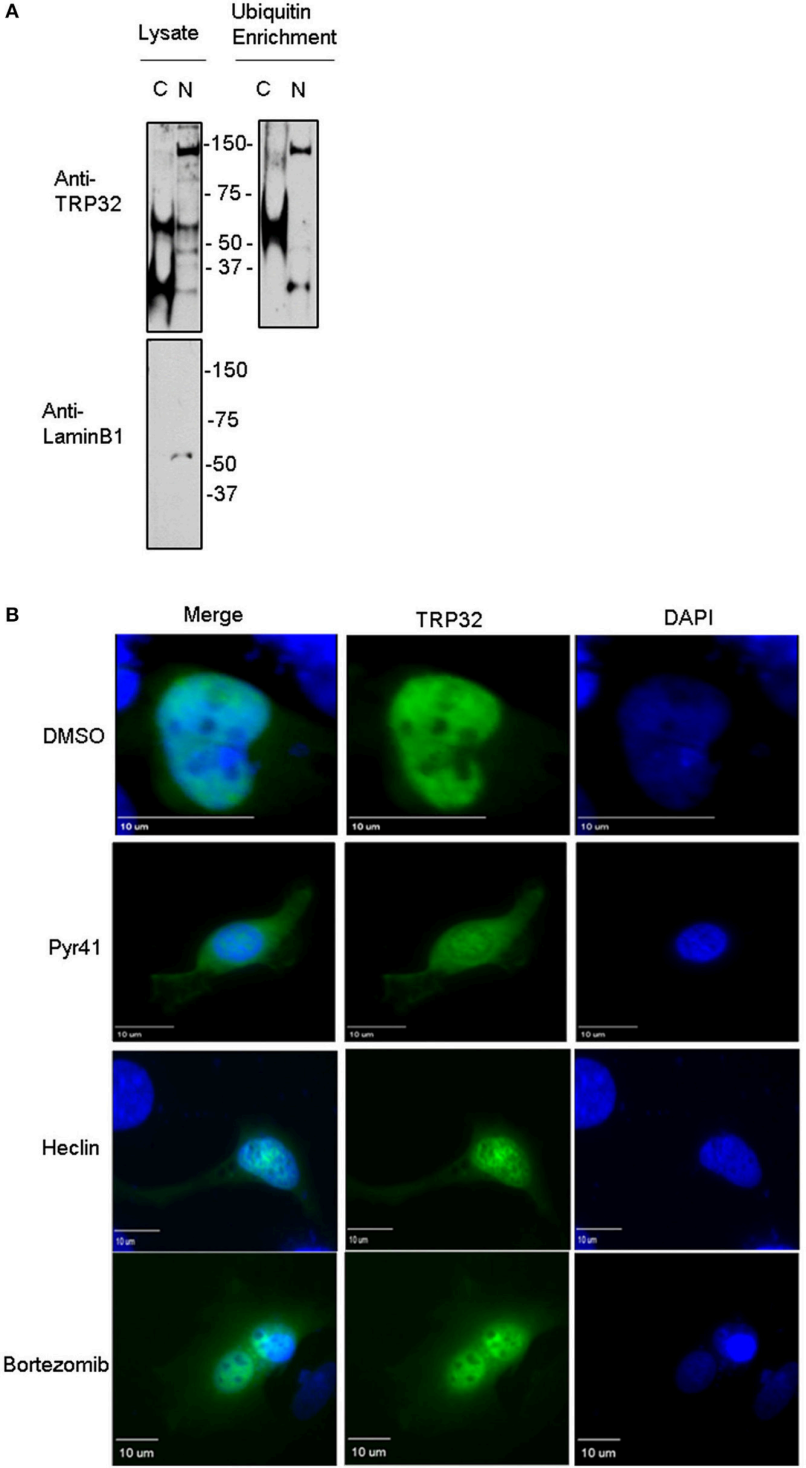
Ubiquitination inhibitors cause altered subcellular localization of GFP-tagged TRP32. **(A)** Polyubiquitinated TRP32 is detected in both the nucleus and cytosol of infected cells. *E. chaffeensis*-infected THP-1 cells were harvested and subject to nuclear cytosolic fractionation before Ub enrichment. Lysate and eluate from Ub enrichment were probed with anti-TRP32 specific antibody by immunoblot. **(B)** HeLa cells were transfected with GFP-tagged TRP32. After 6 h the medium was changed and Pyr41 (50 μM), heclin (25 μM), bortezomib (100 nM) or DMSO (vehicle) were added. At 24 h post-transfection cells were fixed and visualized using fluorescent microscopy. Nuclei are stained blue (DAPI).

**Figure 5:**
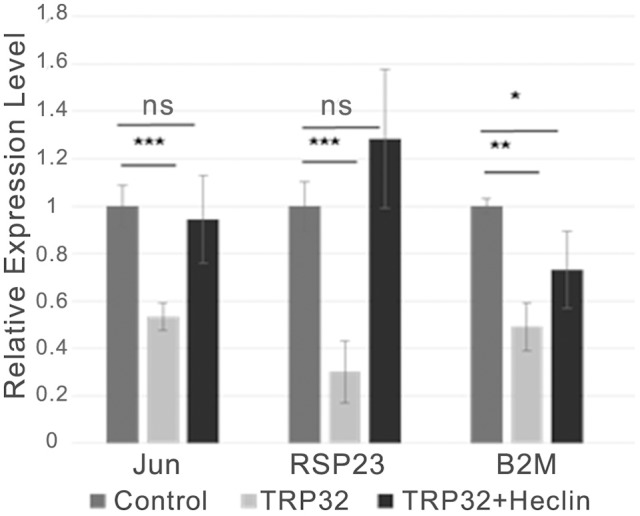
TRP32-mediated transcriptional repression is alleviated by the HECT E3 Ub ligase inhibitor heclin. GFP-TRP32 or a GFP control were transfected into HeLa cells with TRP32-target promoter firefly luciferase expression constructs and *Renilla* luciferase expression vectors in the presence of the HECT E3 Ub ligase inhibitor heclin or vehicle. After 24 h luciferase expression was measured and normalized to constitutive *Renilla* luciferase expression and is presented as fold-change from control. Significance was determined using a Student's *t*-test (^*^p ≤ 0.05, ^**^p ≤ 0.01, ^***^p ≤ 0.001). Graph is representative of at least three experiments.

Because both K63-linked polyubiquitination and monoubiquitination have been linked to regulation of substrate localization, we examined the role of ubiquitination in TRP32 localization within the host cell. First, we examined the localization of polyubiquitinated TRP32. Infected cells were harvested and nuclear cytosolic fractionation performed before applying the lysates to Ub enrichment beads. The resulting eluate was probed with a TRP32-specific antibody. Higher molecular weight TRP32 was enriched in both the cytosolic (~60 kDa) and nuclear (~60 and 150 kDa) fractions. However, different species were found in the different compartments, with ~150-kDa isoform found only in the nuclear fraction (Figure 4A). This suggested that ubiquitination may indeed play a role in TRP32 compartment-specific localization or function. To further address this, we investigated the role of Ub in TRP32 localization by ectopically expressing TRP32 in cells that had been treated with either an E1 inhibitor (Pyr41, pan-ubiquitination inhibitor), a HECT E3 Ub ligase-specific inhibitor (heclin), or a proteasome inhibitor (bortezomib). We found that treatment with Pyr41 resulted in gross alterations in TRP32 localization with the majority of TRP32 occurring in the cytosol and perinuclear regions. Treatment with heclin did not cause major alterations in localization, although subtle differences in subnuclear localization were seen, including increased nuclear speckles and granularity. Neither of these phenotypes were due to accumulation of undegraded cellular protein as treatment with the proteasome inhibitor bortezomib did not alter cellular localization of TRP32 (Figure 4B). In order to test this possibility, we performed a cellular assay using a firefly luciferase reporter expressed under control of the TRP32 target promoters in the presence or absence of these inhibitors (Farris et al., 2016). We found that Pyr41 caused drastic decreases in expression of both the constitutively transcribed *Renilla* luciferase and the firefly luciferase suggesting that this inhibitor broadly dysregulated transcription (data not shown). However, when heclin was used, we found that TRP32-mediated repression of firefly luciferase was removed and that expression returned to levels similar to the control (Figure 5).

**Figure 6:**
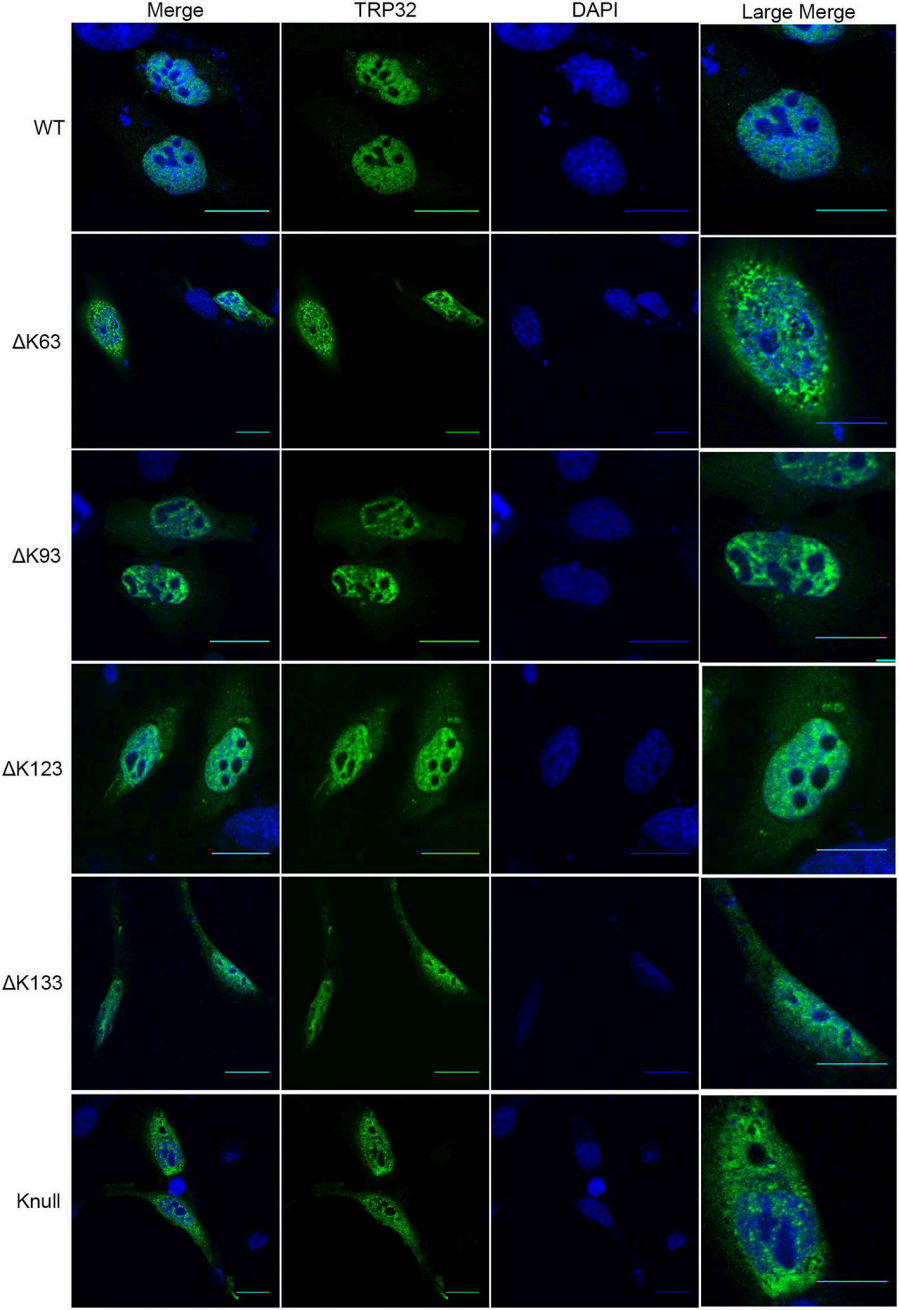
TRP32 lysine mutants demonstrate altered subcellular localization of GFP-tagged TRP32. HeLa cells were transfected with WT and lysine mutant GFP-tagged TRP32. At 24 h post-transfection cells were fixed and visualized using confocal microscopy. Nuclei are stained blue (DAPI).

**Figure 7:**
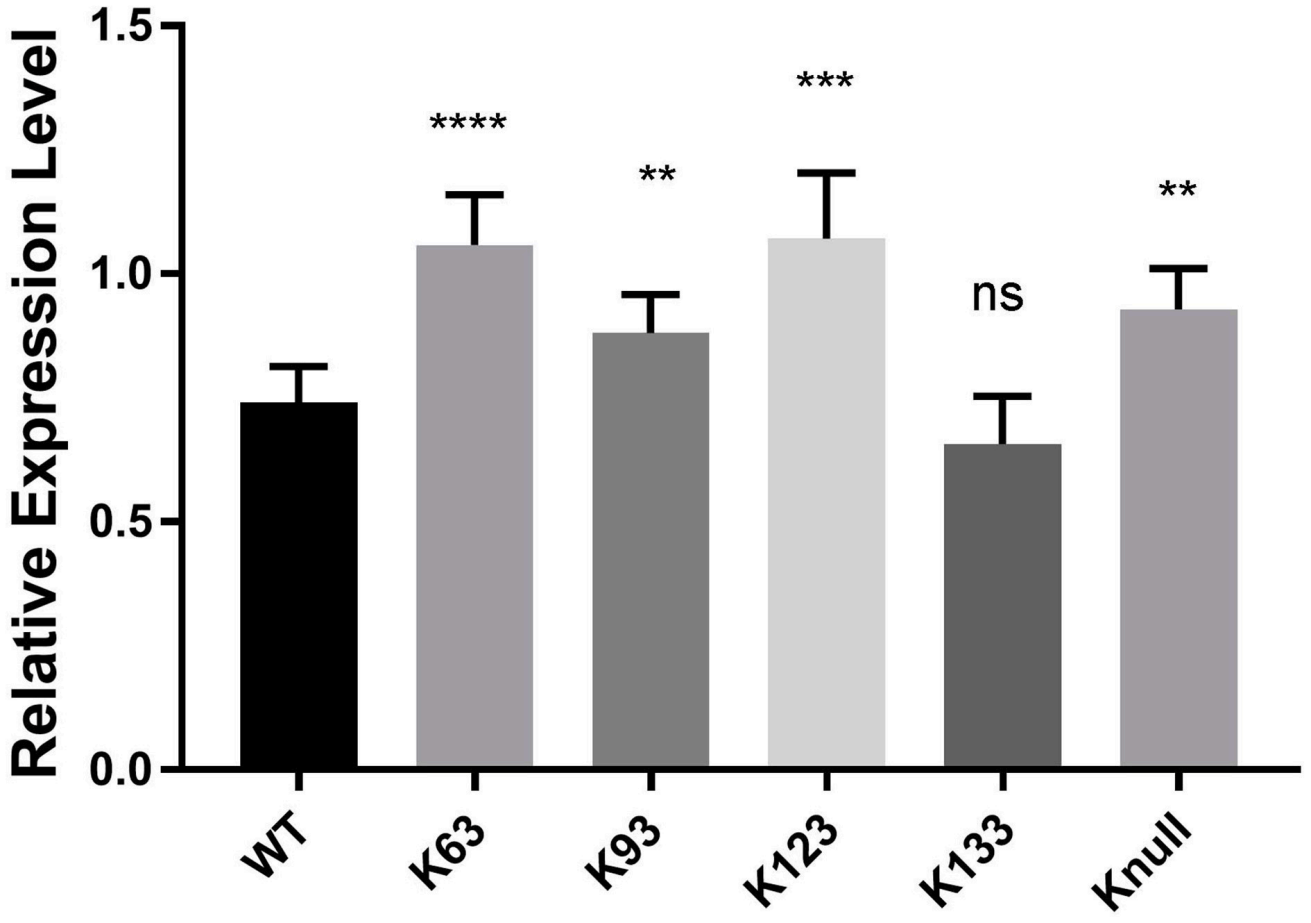
TRP32 lysine mutants exhibit decreased repression of target gene transcription. Wild type and lysine mutant GFP-TRP32, or a GFP control were transfected into HeLa cells with TRP32-target promoter firefly luciferase expression constructs. After 24 h luciferase expression was measured and is presented as fold-change from control. Significance was determined using a Student's *t*-test (^**^p ≤ 0.01, ^***^p ≤ 0.001). Graph is representative of four experiments.

Next, in order to confirm that transcription repression by TRP32 is due to direct ubiquitination of TRP32 and to try to identify a particular lysine residue involved, a series of lysine mutants were created, including a K-null mutant in which all four lysines were deleted. When these mutants were ectopically expressed, the K63, K93, and K123 mutants showed phenotypes characterized by peri-nucleolar rings and puncta, similar to that seen in the heclin treated cells (Figure 6). This phenotype was also observed with the K-null mutant. However, the K133 mutant showed localization similar to WT. Additionally, while the K63, K93, and K123 showed increased protein at the nuclear periphery, the K-null mutant showed much higher levels of cytosolic TRP32. When these mutants were used in a luciferase assay we found that the K-null TRP32 was unable to repress transcription of its target (Figure 7; Figure S1). Transcriptional repression was also relieved by the K63, K93, and the K123 single mutants. Although our *in vitro* Ub array indicated that K93 was not ubiquitinated, *in silico* analysis predicted K93 ubiquitination with high confidence. The cellular localization phenotype of the K93 mutant as well as transcriptional data indicates that it is also ubiquitinated. K133 which does not appear to be ubiquitinated exhibited localization similar to WT, and was able to repress target gene transcription similar to WT TRP32.

## Discussion

The identification of bacterial effectors and defining their function and regulation within the host cell is essential for understanding how obligately intracellular bacteria such as *E. chaffeensis* manipulate the host cell for survival. Previously, we identified TRP32 as a nucleomodulin that regulated transcription of host genes related to cellular differentiation and proliferation (Farris et al., 2016). We have also recently demonstrated that NEDD4L is involved in ubiquitination of *E. chaffeensis* TRP120 (Zhu et al., 2017). In this study, we explored how *E. chaffeensis* exploits the host Ub system to regulate TRP32 localization and function.

Previously, we have shown that TRP32 is a dual function transcription factor (Farris et al., 2016). Although TRP32 possesses putative TADs and was able to activate gene transcription in yeast during infection of a mammalian cell line, the majority of TRP32 targets were downregulated and only a few were upregulated. This was also seen in a luciferase assay using TRP32 gene targets (Farris et al., 2016). Typically, dual function transcription factors that can recruit either coactivators or corepressors function in one of two ways. For some, transcription factor function is promoter-dependent, while for others, function can switch between activation and repression in a signal-dependent manner, typically by phosphorylation, SUMOylation or ubiquitination of the transcription factor (Ross et al., 2002; Boyle and Després, 2010). TRP32 is predicted to be serine/threonine phosphorylated at several sites, and tyrosine ubiquitination has been shown to play a role in TRP32 nuclear localization. Since these modifications may be required for transcription factor function, we focused on the role of Ub, in part because TRP32 has no predicted SUMOylation sites, and SUMOylation was not detected during infection or *in vitro* (data not shown). Additionally, the Ub-proteasome system plays an important role in both positively and negatively regulating several transcription factors. Interestingly, several transcription factors including VP19 and Myc that possess a 9 amino acid TAD (also identified in TRP32), require polyubiquitination for TAD function (Salghetti et al., 2000). Additionally, transcription factor activation is intimately linked to proteasomal degradation in a wide variety of eukaryotic transcription factors (Molinari et al., 1999). Indeed, the proteasome is integral to transcription where it performs both proteolytic and non-proteolytic functions by playing a role in coactivator recruitment, transcription elongation, and histone modification in addition to protein degradation (Geng et al., 2012). The proximity of ubiquitinated lysines to the TRP32 9aa TADs led us to consider that such a mechanism might be at play in TRP32 transcription factor function. However, our data demonstrated that ubiquitination is required for TRP32 repressor function. This may be because TRP32 is not ubiquitinated with K48-linked polyUb chains, but instead with K63-linked chains. Indeed, the TRP32 C-terminus (which lacks lysine residues) was previously shown to interact with a member of the 20S proteasome, PSMB1, which is known to play a role in transcriptional regulation (O'Hara et al., 2013; Yamauchi et al., 2013). Therefore, TRP32 may be able to recruit necessary components of the proteasome independently.

K63-linked Ub has been tied to the function of several transcription factors including IRF1, RORyt, and FOXO family transcription factors (Schisler et al., 2008; Tran et al., 2008; Harikumar et al., 2014; Metcalf et al., 2014; Wang et al., 2015; Zhu et al., 2015; Zeman and Cimprich, 2012). Because K63-linked Ub chains can act as scaffolds for protein-protein interaction, these chains may be required to recruit corepressors or coactivators. There is evidence that K63-linked polyUb chains can be required for the recruitment of both histone deacetylases (HDACs) and members of the transcription initiation complex (Ramakrishna et al., 2011; Wu et al., 2016). However, it remains to be determined if this K63-linked ubiquitination is the most important modification of TRP32 in terms of transcriptional regulation. The inhibitor data with heclin appears to support this conclusion as NEDD4L is known to primarily conjugate K63-linked Ub chains to its targets. However, it also has been shown to conjugate monoUb as well as K6, K11, K26, and K29 atypical chains (Ding et al., 2013; Berndsen and Wolberger, 2014; Michel et al., 2015). Additionally, a recent study reported that one of the reasons that K63-linked chains are not linked to proteasomal degradation may be due to competitive interactions with members of ESCRT0 (Nathan et al., 2013). Although not addressed by this study, other research also implicates K63-linked chains in multivesicular body biogenesis and cargo sorting, suggesting K63-linked polyUb could play a role in TRP32 release from the ehrlichial vacuole or in intracellular trafficking.

Notably, our data suggests that ubiquitination at all three lysine residues (K63, K93, and K123) is required for TRP32 repressor function, as mutation of any one of these three lysine residues resulted in alleviation of TRP32-mediated transcriptional repression. Ubiquitination of these lysine targets were correctly predicted by the *in silico* analysis, and 2 of the 3 were confirmed by the *in vitro* peptide. Whether these lysine residues are modified by multi-monoUb, multi-polyUb, or a mix of the two remains to be determined. Each of these scenarios has been described in the literature and indeed a pattern of complex, multifunctional Ub modifications is typical of many eukaryotic transcription factors and TRP32 seems to be an adept mimic.

We found that TRP32 interacted with, and was ubiquitinated by the human E3 Ub ligase NEDD4L during infection and *in vitro*. We have recently shown NEDD4L interacts and ubiquitinates *E. chaffeensis* effector TRP120 (Zhu et al., 2017). Although TRP32 lacks the canonical NEDD4L PPxY interaction motif, it does contain three similar C-terminal motifs (TPYY, NPYY, and TPDY) that may mediate interactions with NEDD4L. Other NEDD family members have been shown to interact with (pS/pT) PxY motifs in addition to the canonical PPxY motif (Lu et al., 1999). Additionally, several known NEDD4L targets lack the PPxY motif completely and interact with NEDD4L via adaptor proteins, which may be the case with TRP32 (Mund and Pelham, 2009; de Groot et al., 2014).

Although NEDD4L has not previously been studied in the context of other bacteria, it has been linked to viral infections including HIV infection where K63-linked ubiquitination of the viral protein Gag by NEDD4L plays an important role in viral budding and in enterovirus 71 where it may function via altering IFN-β production (Chung et al., 2008; Kuo et al., 2015). In uninfected human cells, NEDD4L has been most commonly studied in the context of the regulation of membrane proteins such as sodium and potassium channels which are directed to recycling endosomes by NEDD4L (Scheffner and Kumar, 2014). However, NEDD4L has also been shown to be an important regulator of the TGF-β and Wnt signaling pathway via ubiquitination of SMADs and Disheveled proteins, respectively (Gao et al., 2009; Ding et al., 2013). Especially interesting in the context of *Ehrlichia* infection is that NEDD4L is phosphorylated and activated by JNK in a WNT5a-dependent manner (Ding et al., 2013). Previously, we have shown that both canonical and non-canonical Wnt signaling are vitally important for successful *E. chaffeensis* infection, and WNT5a knockdown results in significantly decreased ehrlichial infection (Luo et al., 2015). It is likely that WNT5a activation of NEDD4L plays a role in licensing TRP32 transcription factor function. It may do this by regulating TRP32s interactions with host proteins as was demonstrated with TRP120 (Zhu et al., 2017).

During infection, we determined that TRP32 interacts with NEDD4L and can be ubiquitinated by NEDD4L *in vitro*. Multiple mono- and polyubiquitinated forms of TRP32 were detected during infection, including some which did not completely disappear when cells were treated with the NEDD4L inhibitor. Further, we found that treatment with heclin both impaired the ability of TRP32 to repress target gene transcription and altered its subnuclear localization, and that mutation of specific TRP32 lysine residues mimicked these phenotypes. It is likely that ubiquitination may serve multiple functions in regulating TRP32 and that other Ub ligases are required in addition to NEDD4L. Indeed, TRP32 is known to react with the putative RING-type E3 Ub ligase Roquin (Luo and McBride, 2012). Future directions include identifying the mechanism of altered TRP32 transcriptional function mediated by ubiquitination, fully characterizing the Ub modifications that occur on the various TRP32 lysine residues, and examining the potential crosstalk between these various Ub modifications and other TRP32 PTMs.

## Author contributions

TF: Plan research, perform experiments, manuscript prep, and editing. BZ: Plan research, perform experiments. JW: Manuscript prep and editing. JM: Plan research, manuscript prep and editing.

### Conflict of interest statement

The authors declare that the research was conducted in the absence of any commercial or financial relationships that could be construed as a potential conflict of interest.

## Supplementary material

Figure S1 Expression levels of TRP32 wild type and lysine mutant expression constructs in HeLa cells.

## References

[B1] Anderson D. M. Frank D. W. (2012). Five mechanisms of manipulation by bacterial effectors: a ubiquitous theme. PLoS Pathog. 8:e1002823. 10.1371/journal.ppat.1002823 22927812 PMC3426537

[B2] Ashida H. Kim M. Sasakawa C. (2014). Exploitation of the host ubiquitin system by human bacterial pathogens. Nat. Rev. Micro. 12, 399–413. 10.1038/nrmicro3259 24801936

[B3] Berndsen C. E. Wolberger C. (2014). New insights into ubiquitin E3 ligase mechanism. Nat. Struct. Mol. Biol. 21, 301–307. 10.1038/nsmb.2780 24699078

[B4] Boyle P. Després C. (2010). Dual-function transcription factors and their entourage: unique and unifying themes governing two pathogenesis-related genes. Plant Signal. Behav. 5, 629–634. 10.4161/psb.5.6.11570 20383056 PMC3001550

[B5] Chung H.-Y. Morita E. von Schwedler U. Müller B. Kräusslich H.-G. Sundquist W. I. (2008). NEDD4L overexpression rescues the release and infectivity of human immunodeficiency virus Type 1 constructs lacking PTAP and YPXL late domains. J. Virol. 82, 4884–4897. 10.1128/JVI.02667-07 18321968 PMC2346761

[B6] Collins C. A. Brown E. J. (2010). Cytosol as battleground: ubiquitin as a weapon for both host and pathogen. Trends Cell Biol. 20, 205–213. 10.1016/j.tcb.2010.01.002 20129784

[B7] de Groot T. Trimpert C. Wesche D. Wong V. van den Berg D. Stagljar I. (2014). NDFIP1: the missing adaptor for aquaporin-2 regulation by NEDD4 and NEDD4L (LB723). FASEB J. 28(Suppl. 1):LB723 10.1096/fj.1530-6860

[B8] Ding Y. Zhang Y. Xu C. Tao Q. H. Chen Y. G. (2013). HECT Domain-containing E3 Ubiquitin Ligase NEDD4L negatively regulates Wnt signaling by targeting dishevelled for proteasomal degradation. J. Biol. Chem. 288, 8289–8298. 10.1074/jbc.M112.433185 23396981 PMC3605647

[B9] Dunphy P. S. Luo T. McBride J. W. (2014). *Ehrlichia chaffeensis* exploits host SUMOylation pathways to mediate effector-host interactions and promote intracellular survival. Infect. Immun. 82, 4154-4168. 10.1128/IAI.01984-14 25047847 PMC4187855

[B10] Farris T. R. Dunphy P. S. Zhu B. Kibler C. E. McBride J. W. (2016). *Ehrlichia chaffeensis* TRP32 is a nucleomodulin that directly regulates expression of host genes governing differentiation and prolimferation. Infect. Immun. 84, 3184–3194. 10.1128/IAI.00657-16 PMC5067751 27572329

[B11] Gao S. Alarcón C. Sapkota G. Rahman S. Chen P.-Y. Goerner N.. (2009). Ubiquitin Ligase Nedd4L targets activated Smad2/3 to limit TGF-β signaling. Mol. Cell 36, 457–468. 10.1016/j.molcel.2009.09.043 19917253 PMC2796330

[B12] Geng F. Wenzel S. Tansey W. P. (2012). Ubiquitin and Proteasomes in transcription. Annu. Rev. Biochem. 81, 177–201. 10.1146/annurev-biochem-052110-120012 22404630 PMC3637986

[B13] Harikumar K. B. Yester J. W. Surace M. J. Oyeniran C. Price M. M. Huang W.-C. (2014). K63-linked polyubiquitylation of IRF1 transcription factor is essential for IL-1-induced CCL5 and CXCL10 chemokine production. Nat. Immunol. 15, 231–238. 10.1038/ni.2810 24464131 PMC3976678

[B14] Komander D. (2009). The emerging complexity of protein ubiquitination. Biochem. Soc. Trans. 37(Pt 5), 937–953. 10.1042/BST0370937 19754430

[B15] Kuo R.-L. Lin Y.-H. Wang R. Y.-L. Hsu C.-W. Chiu Y.-T. Huang H.-I.. (2015). Proteomics analysis of EV71-infected cells reveals the involvement of host protein NEDD4L in EV71 replication. J. Proteome Res. 14, 1818–1830. 10.1021/pr501199h 25785312

[B16] Kuriakose J. A. Zhang X. Luo T. McBride J. W. (2012). Molecular basis of antibody mediated immunity against *Ehrlichia chaffeensis* involves species-specific linear epitopes in tandem repeat proteins. Microbes Infect. 14, 1054–1063. 10.1016/j.micinf.2012.05.012 22658957 PMC3445803

[B17] Lina T. T. Dunphy P. S. Luo T. McBride J. W. (2016a). *Ehrlichia chaffeensis* TRP120 Activates canonical Notch signaling to downregulate TLR2/4 expression and promote intracellular survival. MBio 7:e00672-16. 10.1128/mBio.00672-16 27381289 PMC4958247

[B18] Lina T. T. Farris T. Luo T. Mitra S. Zhu B. McBride J. W. (2016b). Hacker within! *Ehrlichia chaffeensis* effector driven phagocyte reprogramming strategy. Front. Cell. Infect. Microbiol. 6:58. 10.3389/fcimb.2016.00058 27303657 PMC4885862

[B19] Lu P.-J. Zhou X. Z. Shen M. Lu K. P. (1999). Function of WW Domains as phosphoserine- or phosphothreonine-binding modules. Science 283, 1325-1328. 10.1126/science.283.5406.1325 10037602

[B20] Luo T. Dunphy P. S. Lina T. T. McBride J. W. (2015). *Ehrlichia chaffeensis* exploits canonical and noncanonical host Wnt signaling pathways to stimulate phagocytosis and promote intracellular survival. Infect. Immun. 84, 686–700. 10.1128/IAI.01289-15 26712203 PMC4771358

[B21] Luo T. McBride J. W. (2012). *Ehrlichia chaffeensis* TRP32 interacts with host cell targets that influence intracellular survival. Infect. Immun. 80, 2297–2306. 10.1128/IAI.00154-12 22547548 PMC3416477

[B22] Luo T. Zhang X. Wakeel A. Popov V. L. McBride J. W. (2008). A variable-length PCR target protein of *Ehrlichia chaffeensis* contains major species-specific antibody epitopes in acidic serine-rich tandem repeats. Infect. Immun. 76, 1572–1580. 10.1128/IAI.01466-07 18212082 PMC2292866

[B23] McBride J. W. Zhang X. Wakeel A. Kuriakose J. A. (2011). Tyrosine-phosphorylated *Ehrlichia chaffeensis* and *Ehrlichia canis* tandem repeat orthologs contain a major continuous cross-reactive antibody epitope in lysine-rich repeats. Infect. Immun. 79, 3178–3187. 10.1128/IAI.01347-10 21606187 PMC3147547

[B24] McLean C. Y. Bristor D. Hiller M. Clarke S. L. Schaar B. T. Lowe C. B.. (2010). GREAT improves functional interpretation of cis-regulatory regions. Nat. Biotechnol. 28, 495–501. 10.1038/nbt.1630 20436461 PMC4840234

[B25] Metcalf J. L. Bradshaw P. S. Komosa M. Greer S. N. Stephen Meyn M. Ohh M. (2014). K63-Ubiquitylation of VHL by SOCS1 mediates DNA double-strand break repair. Oncogene 33, 1055–1065. 10.1038/onc.2013.22 23455319

[B26] Metzger M. B. Hristova V. A. Weissman A. M. (2012). HECT and RING finger families of E3 ubiquitin ligases at a glance. J. Cell Sci. 125, 531–537. 10.1242/jcs.091777 22389392 PMC3381717

[B27] Michel M. A. Elliott P. R. Swatek K. N. Simicek M. Pruneda J. N. Wagstaff J. L.. (2015). Assembly and specific recognition of k29- and k33-linked polyubiquitin. Mol. Cell 58, 95–109. 10.1016/j.molcel.2015.01.042 25752577 PMC4386031

[B28] Molinari E. Gilman M. Natesan S. (1999). Proteasome-mediated degradation of transcriptional activators correlates with activation domain potency *in vivo* EMBO J. 18, 6439–6447. 10562555 10.1093/emboj/18.22.6439 PMC1171706

[B29] Mund T. Pelham H. R. B. (2009). Control of the activity of WW-HECT domain E3 ubiquitin ligases by NDFIP proteins. EMBO Rep. 10, 501-507. 10.1038/embor.2009.30 19343052 PMC2680872

[B30] Nathan J. A. Kim H. T. Ting L. Gygi S. P. Goldberg A. L. (2013). Why do cell proteins linked to K63-polyubiquitin chains not associate with proteasomes? EMBO J. 32, 552–565. 10.1038/emboj.2012.354 23314748 PMC3579138

[B31] O'Hara A. Howarth A. Varro A. Dimaline R. (2013). The role of proteasome beta subunits in gastrin-mediated transcription of plasminogen activator inhibitor-2 and regenerating protein1. PLoS ONE 8:e59913. 10.1371/journal.pone.0059913 23544109 PMC3609805

[B32] Patel J. C. Hueffer K. Lam T. T. Galán J. E. (2009). Diversification of a Salmonella virulence protein function by ubiquitin-dependent differential localization. Cell 137, 283–294. 10.1016/j.cell.2009.01.056 19379694 PMC2673707

[B33] Popa C. M. Tabuchi M. Valls M. (2016). Modification of bacterial effector proteins inside Eukaryotic host cells. Front. Cell. Infect. Microbiol. 6:73. 10.3389/fcimb.2016.00073 27489796 PMC4951486

[B34] Radivojac P. Vacic V. Haynes C. Cocklin R. R. Mohan A. Heyen J. W.. (2010). Identification, analysis, and prediction of protein ubiquitination sites. Proteins 78, 365–380. 10.1002/prot.22555 19722269 PMC3006176

[B35] Ramakrishna S. Suresh B. Lee E.-J. Lee H.-J. Ahn W.-S. Baek K.-H. (2011). Lys-63-specific Deubiquitination of SDS3 by USP17 Regulates HDAC activity. J. Biol. Chem. 286, 10505–10514. 10.1074/jbc.M110.162321 21239494 PMC3060504

[B36] Ravikumar V. Jers C. Mijakovic I. (2015). Elucidating host–pathogen interactions based on post-translational modifications using proteomics approaches. Front. Microbiol. 6:1313. 10.3389/fmicb.2015.01312 26635773 PMC4653285

[B37] Ribet D. Cossart P. (2010). Post-translational modifications in host cells during bacterial infection. FEBS Lett. 584, 2748–2758. 10.1016/j.febslet.2010.05.012 20493189

[B38] Ross S. Best J. L. Zon L. I. Gill G. (2002). SUMO-1 Modification Represses Sp3 transcriptional activation and modulates its subnuclear localization. Mol. Cell 10, 831–842. 10.1016/S1097-2765(02)00682-2 12419227

[B39] Salghetti S. E. Muratani M. Wijnen H. Futcher B. Tansey W. P. (2000). Functional overlap of sequences that activate transcription and signal ubiquitin-mediated proteolysis. Proc. Natl. Acad. Sci. U.S.A. 97, 3118–3123. 10.1073/pnas.97.7.3118 10706616 PMC16202

[B40] Salmena L. Pandolfi P. P. (2007). Changing venues for tumour suppression: balancing destruction and localization by monoubiquitylation. Nat. Rev. Cancer 7, 409–413. 10.1038/nrc2145 17508027

[B41] Scheffner M. Kumar S. (2014). Mammalian HECT ubiquitin-protein ligases: biological and pathophysiological aspects. Biochim. Biophys. Acta 1843, 61–74. 10.1016/j.bbamcr.2013.03.024 23545411

[B42] Schindelin J. Arganda-Carreras I. Frise E. Kaynig V. Longair M. Pietzsch T.. (2012). Fiji: an open-source platform for biological-image analysis. Nat. Methods 9, 676–682. 10.1038/nmeth.2019 22743772 PMC3855844

[B43] Schisler J. C. Willis M. S. Patterson C. (2008). You spin me round: mafBx/Atrogin-1 feeds forward on FOXO transcription factors (like a record). Cell Cycle 7, 440–443. 10.4161/cc.7.4.5451 18235241

[B44] Shirai A. Matsuyama A. Yashiroda Y. Hashimoto A. Kawamura Y. Arai R.. (2008). Global analysis of gel mobility of proteins and its use in target identification. J. Biol. Chem. 283, 10745–10752. 10.1074/jbc.M709211200 18292091

[B45] Thomas M. Holden D. W. (2009). Ubiquitination - a bacterial effector's ticket to ride. Cell Host Microbe 5, 309–311. 10.1016/j.chom.2009.03.010 19380107

[B46] Tran H. Hamada F. Schwarz-Romond T. Bienz M. (2008). Trabid, a new positive regulator of Wnt-induced transcription with preference for binding and cleaving K63-linked ubiquitin chains. Genes Dev. 22, 528–542. 10.1101/gad.463208 18281465 PMC2238673

[B47] Wakeel A. den Dulk-Ras A. Hooykaas P. J. McBride J. W. (2011). *Ehrlichia chaffeensis* tandem repeat proteins and Ank200 are type 1 secretion system substrates related to the repeats-in-toxin exoprotein family. Front. Cell. Infect. Microbiol. 1:22. 10.3389/fcimb.2011.00022 22919588 PMC3417381

[B48] Wang X. Yang J. Han L. Zhao K. Wu Q. Bao L. (2015). TRAF5-mediated K63-linked polyubiquitination play essential role in positive regulation of RORγt on promoting IL-17A expression. J. Biol. Chem. 290, 29086-29094. 10.1074/jbc.M115.664573 26453305 PMC4661420

[B49] Wu H.-T. Kuo Y.-C. Hung J.-J. Huang C.-H. Chen W.-Y. Chou T.-Y.. (2016). K63-polyubiquitinated HAUSP deubiquitinates HIF-1α and dictates H3K56 acetylation promoting hypoxia-induced tumour progression. Nat. Commun. 7:13644. 10.1038/ncomms13644 27934968 PMC5155157

[B50] Yamauchi J. Sekiguchi M. Shirai T. Yamada M. Ishimi Y. (2013). Role of nuclear localization of PSMB1 in transcriptional activation. Biosci. Biotechnol. Biochem. 77, 1785–1787. 10.1271/bbb.130290 23924720

[B51] Zeman M. K. Cimprich K. A. (2012). Finally, Polyubiquitinated PCNA gets recognized. Mol. Cell 47, 333–334. 10.1016/j.molcel.2012.07.024 22883622

[B52] Zhu B. Das S. Mitra S. Farris T. R. McBride J. W. (2017). *Ehrlichia chaffeensis* TRP120 moonlights as a HECT E3 ligase involved in self and host ubiquitination to influence protein interactions and stability for intracellular survival. Infect. Immun. 85:e00290-17. 10.1128/IAI.00290-17 28630068 PMC5563569

[B53] Zhu B. Farris T. Milligan S. Chen H. S. Zhu R. J. Hong A.. (2016). Rapid identification of ubiquitination and SUMOylation target sites by microfluidic peptide array. Biochem. Biophys. Rep. 5, 430–438. 10.1016/j.bbrep.2016.02.003 27047992 PMC4817105

[B54] Zhu B. Kuriakose J. A. Luo T. Ballesteros E. Gupta S. Fofanov Y.. (2011). *Ehrlichia chaffeensis* TRP120 binds a G+C-rich motif in host cell DNA and exhibits eukaryotic transcriptional activator function. Infect. Immun. 79, 4370–4381. 10.1128/IAI.05422-11 21859854 PMC3257946

[B55] Zhu B. Yan K. Li L. Lin M. Zhang S. He Q.. (2015). K63-linked ubiquitination of FANCG is required for its association with the Rap80-BRCA1 complex to modulate homologous recombination repair of DNA interstand crosslinks. Oncogene 34, 2867–2878. 10.1038/onc.2014.229 25132264

