# Supplementary figures and images for: Ehrlichia chaffeensis TRP32 Nucleomodulin Function and Localization Is Regulated by NEDD4L-Mediated Ubiquitination
Source: Front Cell Infect Microbiol. 2018 Jan 11;7:534. doi: 10.3389/fcimb.2017.00534 (PMC5768648; doi:10.3389/fcimb.2017.00534)

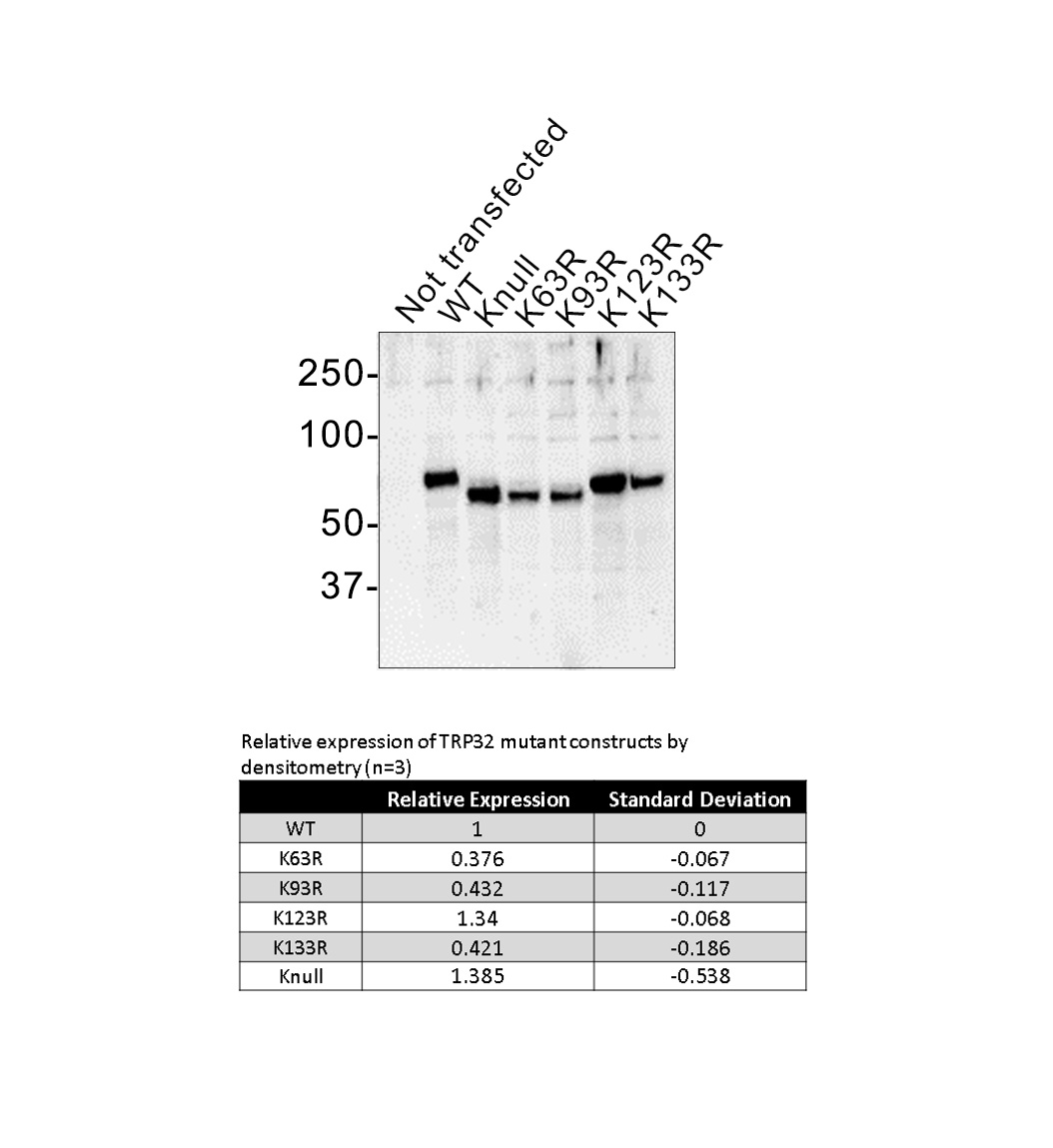

Supplement: Figure S1 — Expression levels of TRP32 wild type and lysine mutant expression constructs in HeLa cells. [file Image1.JPEG]
